# Successes and failures: what did we learn from recent first-line treatment immunotherapy trials in non-small cell lung cancer?

**DOI:** 10.1186/s12916-017-0819-3

**Published:** 2017-03-13

**Authors:** Jordi Remon, Benjamin Besse, Jean-Charles Soria

**Affiliations:** 1 Cancer Medicine Department, Gustave Roussy, Villejuif, France; 2 Medical Oncology Department, Hospital Vall d’Hebron, Barcelona, Spain; 3 University Paris-Sud, Orsay, France

**Keywords:** Immunotherapy, Pembrolizumab, Nivolumab, PD-1, PD-L1, First-line, Non-small cell lung cancer

## Abstract

The immune checkpoint inhibitors have significantly modified the therapeutic landscape of advanced non-small cell lung cancer in second-line and, more recently, first-line settings. Because of the superior outcome with pembrolizumab as an upfront strategy, PD-L1 status should now be considered a new reflex biomarker for guiding first-line treatment in patients with advanced non-small cell lung cancer. Improved responses have also been reported with the combination of immune checkpoint inhibitors and chemotherapy as the first-line treatment; however, this strategy has not yet been validated by phase III trial data and its interplay with PD-L1 status still requires clarification.

In this manuscript we review the contradictory results of recent phase III trials with immune checkpoint inhibitors in the first-line setting, the potential reasons for discrepancies, and some of the remaining open questions related to the positioning of immune checkpoint inhibitors in the first-line setting of non-small cell lung cancer.

## Introduction

First-line platinum-based chemotherapy is the standard of care in the majority of patients with advanced non-small cell lung cancer (NSCLC) without comorbidities and with optimal performance status (PS) [1]. This excludes patients with oncogenic driver alterations, such as the epidermal growth factor receptor (*EGFR*) mutation (in almost 50% of patients of Asian ethnicity compared to 15% in the Caucasian population [2]) or the anaplastic lymphoma kinase (*ALK*) re-arrangement (in 5% patients independently of ethnicity [3]) that can be treated with tyrosine kinase inhibitors. However, even in the era of maintenance therapy, platinum-based chemotherapy results in a median progression-free survival (PFS) of ~6 months and a response rate (RR) of ~30% [1]. Therefore, significant advances are eagerly awaited.

A deeper understanding of tumor-immune interactions and development of immune checkpoint inhibitors has dramatically changed the therapeutic landscape of NSCLC and other malignancies. The immune system recognizes and is poised to eliminate cancer [4]. Immune checkpoints refer to a variety of inhibitory pathways that are crucial for regulating the duration and amplitude of physiological immune responses in peripheral tissues in order to minimize collateral tissue damage [5]. However, these immune checkpoint pathways can be co-opted by cancer cells, circumventing immune destruction [4], which is a hallmark of cancer [6]. In NSCLC, expression of programmed death ligand-1 (PD-L1, B7-H1) reflects an immune-active microenvironment and is a mechanism for evading elimination by the immune system [7]. Exhausted T-cells in the microenvironment show overexpression of programmed cell death protein 1 (PD-1), which binds to PD-L1 and decreases effector cytokine production and cytolytic activity, leading to the failure of cancer elimination [8]. This knowledge has prompted the development of immune checkpoint inhibitors – different monoclonal antibodies that bind either to PD-1 or PD-L1 and hamper immune evasion – as new treatment strategies in advanced NSCLC.

Four randomized phase III trials have reported a statistically significant improvement in overall survival (OS) with immune checkpoint inhibitors compared with docetaxel in patients with platinum-refractory advanced NSCLC: the CheckMate017 trial [9] in patients with squamous NSCLC; the CheckMate057 [10] in patients with non-squamous NSCLC (both trials testing nivolumab, a monoclonal-antibody anti-PD-1); the KEYNOTE-010 phase II/III trial [11] with pembrolizumab, which also binds to PD-1 (inclusion restricted to patients with at least 1% PD-L1 expression on tumor cells); and the OAK trial [12] with atezolizumab, a monoclonal antibody against PD-L1. Of note, in the KEYNOTE 010 study [11], the magnitude of benefit with pembrolizumab correlated with PD-L1 expression, with increased benefit seen in patients with tumors with strong PD-L1 expression, defined as expression on at least 50% of tumor cells, regardless of the staining intensity with the 22C3 clone [11]. On the basis of these trials, the US Food and Drug Administration (FDA) and the European Medicines Agency (EMA) have approved nivolumab and pembrolizumab as second-line therapy, the latter restricted to tumors expressing PD-L1. The FDA has also recently approved atezolizumab for the management of previously treated patients with advanced NSCLC. In the absence of head-to-head comparisons or clear biological differences between these agents, and no significant differences in toxicity profile (except a slight increase in immune-related adverse events (AEs) and pneumonitis with anti-PD-1 inhibitors) [13], it is not possible to recommend one treatment over another.

The 3-year OS of patients with advanced NSCLC treated with these drugs after failure with platinum-based chemotherapy is approaching 20% [14]. This response, along with an improved safety profile, has prompted increasing interest in testing these agents in the first-line setting.

## Anti-PD-1 antibodies as single agent in patients with PD-L1-positive NSCLC

### Pembrolizumab

The phase III KEYNOTE 024 trial comprises patients with advanced and strongly PD-L1-positive NSCLC [15]. A total of 1942 patients were screened for enrolment; 1653 had evaluable samples, and 500 (30.2%) of patients had tumors with PD-L1 expression ≥50%. A total of 305 patients who met inclusion criteria were randomized to pembrolizumab (200 mg every 3 weeks for up to 35 cycles or until documented progressive disease) versus four to six cycles of standard of care platinum-based chemotherapy (platinum/pemetrexed, platinum/gemcitabine, or carboplatin/paclitaxel) as first-line treatment. Pemetrexed maintenance therapy was received by 30% of patients with non-squamous histology. In addition, 43.7% of patients in the control arm crossed over per protocol to pembrolizumab upon disease progression. Patients were excluded from the trial if they were harboring *EGFR* mutations or *ALK* translocations, had an Eastern Cooperative Oncology Group (ECOG) performance status ≥2, had untreated brain metastasis, or were receiving any dose of oral steroids for an autoimmune disease. The primary endpoint of the trial was the median PFS. Compared with standard first-line platinum-based chemotherapy, pembrolizumab significantly improved the primary endpoint from 6.0 months to 10.3 months (hazard ratio [HR] 0.50, 95% confidence interval [CI] 0.37–0.68, *p* < 0.001). The RR according to Response Evaluation Criteria In Solid Tumors (RECIST; 44.8% versus 27.8%, *p* < 0.001) and OS (not reached in both arms, HR 0.60, 95% CI 0.41–0.89, *p* = 0.005) were also improved, with 1-year OS of 70% versus 54% [15]. The benefit of pembrolizumab with respect to PFS was evident in all subgroups examined according to gender, age, histology, smoking status, and brain metastases at baseline. However, the benefit was lower in female and never-smoker patients (probably related to the lower mutational load in this population [16]), and the greatest benefit to PFS was observed in patients with squamous histology (HR 0.35, 95% CI 0.17–0.71). Grade 3, 4, or 5 treatment-related AEs also favored pembrolizumab (26.6% versus 53.3%). The incidence of grade 3–4 immune-mediated AEs was 9.7% with pembrolizumab [15]. Pembrolizumab had a clinically meaningful improvement in quality of life compared to platinum-based chemotherapy [17] (Table 1).

**Table 1:** Immune checkpoint inhibitors in first-line treatment in advanced non-small cell lung cancer patients

Study	Phase	*n*	RR (%)	PFS (months)	OS (months)	AEs ≥ grade 3 (%)
KEYNOTE 024 [15] Pembrolizumab vs. CT	III	305 PD-L1 ≥ 50%*	44.8 vs. 27.8 *p* < 0.001	10.3 vs. 6.0 HR 0.50, *p* < 0.001	HR 0.60, *p* = 0.005 1-year OS: 70% vs. 54%,	26.6 vs. 53.3
CheckMate 026 [31] Nivolumab vs*.* CT	III	423 PD-L1 ≥ 5%**	26.1 vs. 33.5	4.2 vs. 5.9 HR 1.15, *p* = 0.251	HR 1.02 14.4 vs. 13.2 months	17.6 vs. 50.6
KEYNOTE 021 [39] Pembrolizumab + CT vs. CT	II	123	55 vs. 29 p = 0.0016	13.0 vs. 8.9 HR 0.53, *p* = 0.0102	HR 0.90, *p* = 0.39	39 vs. 26%
CheckMate 012 [42] Nivolumab + Ipilimumab/12 weeks until disease progression or toxicity Nivolumab + Ipilimumab/6 weeks until disease progression or toxicity	I	38 40	47 38	8.1 3.9	Not calculated	37 33

*Abbreviations: CT* chemotherapy, *RR* Response rate, *PFS* Progression-free survival, *OS* Overall survival, *AEs* Adverse events. *Expression in ≥50% of tumor cells, regardless of the staining intensity with the 22C3 clone. **Tumor cell membrane staining any intensity >1% with the 28–8 clone Epitomics

The magnitude of benefit in the control arm was consistent with historic controls [18], suggesting that pembrolizumab efficacy is not overestimated for an ineffective control arm. However, it is unknown whether the survival benefit was because pembrolizumab treatment is intrinsically more potent as a first-line treatment or because crossover was limited to <50% of the patients in the control arm. Indeed, trials in patients with *EGFR*-mutant or *ALK*-rearranged NSCLC have had much higher rates of crossover from chemotherapy to personalized treatment after platinum-based chemotherapy progression (65% in *EGFR*-mutant [19] and 70% in *ALK-*positive populations [20]), leading to a lack of survival differences between treatment arms. The clear benefit for OS could also be due to a potentially lower efficacy of pembrolizumab in platinum-pretreated patients than in chemonaïve patients, whereas, in the same settings, targeted therapies yield the same benefit [21, 22].

In the KEYNOTE 024 trial, 11.7% of patients in the pembrolizumab arm had previously treated brain metastases at baseline. The PFS benefit in this subgroup was similar to those patients without brain metastases at baseline (HR 0.55 versus HR 0.50). The efficacy of pembrolizumab in patients with PD-L1 positive (>1%) NSCLC with untreated or progressive asymptomatic brain metastases between 5 and 20 mm of diameter has also recently been tested in a phase II trial that reported a cerebral response rate of 33%. The median duration of confirmed brain responses was 6 months [23]. Approximately 17% of NSCLC patients have brain metastases at baseline [24]. In our opinion, supra-tentorial asymptomatic brain metastases should not be considered exclusion criteria for immune checkpoint inhibitor treatment. The risk of brain metastases increases over time owing to the prolonged survival of patients with advanced NSCLC [25]. Therefore, further investigations are needed to determine optimal treatment combinations with brain radiotherapy, sequences of treatment, and safety [26].

Globally, pembrolizumab results from KEYNOTE 024 [15] were consistent with the efficacy observed in the KEYNOTE 001 [27] study in the subgroup of chemonaïve patients. The FDA approved pembrolizumab in the first-line setting in this population on 24 October 2016, and on 15 December 2016, the EMA Committee for Medicinal Products for Human Use also approved pembrolizumab as monotherapy in the first-line setting of metastatic NSCLC in adults whose tumors express PD-L1 in a tumor proportion score (TPS) ≥50% and who have no *EGFR*- or *ALK*-positive tumor mutations. The efficacy of pembrolizumab as a first-line treatment in NSCLC patients with PD-L1 expression <50% remains unknown. The ongoing phase III KEYNOTE 042 study (NCT02220894) will assess the survival benefit of pembrolizumab over standard first-line platinum-based chemotherapy in treatment-naïve patients who have tumors with ≥1% PD-L1 positivity. Stratification according to PD-L1 expression (strong [≥50%] versus weak [1–49%]) will be performed in the study.

Among the 30% of patients whose tumors express PD-L1 with a TPS ≥ 50%, other clinical exclusion criteria limit the extended use of pembrolizumab in the first-line setting, for example exclusion of patients considered unfit or with poor PS (representing almost 34% of NSCLC patients in contemporary cohorts [28]), patients with *EGFR*-mutant and *ALK*-rearranged tumors (approximately 17% of adenocarcinoma lung cancers in Caucasian populations [29]), and the absence of steroids or autoimmune disorders (13.5% of lung cancer patients [30]). As such, the pool of patients eligible for upfront pembrolizumab is certainly not 30% of all chemonaïve patients with NSCLC (which represents the percentage of frontline patients whose tumors express PD-L1 with a TPS ≥ 50%), but probably closer to 10% of them. This pool clearly needs to be enlarged.

Moreover, the turnaround time from patient selection to treatment, based on PD-L1 expression, is not reported in KEYNOTE 024 but is expected to be frequently longer than one month. There is a high probability that patients with relatively indolent disease were favored for inclusion in the study, adding another bias compared to routine practice. Patients with a poorer prognosis need to be explored, such as in the ongoing phase II trial NCT02879617 evaluating first-line durvalumab in PS2 patients with advanced NSCLC.

### Nivolumab

The phase III CheckMate 026 trial tested the efficacy of nivolumab compared to standard first-line chemotherapy (platinum/pemetrexed, platinum/gemcitabine, or carboplatin/paclitaxel) in 423 patients with PD-L1-positive (≥5% of expression by 28–8 clone) advanced NSCLC [31]. Patients harboring *EGFR* mutations or *ALK* translocations were ineligible. Patients with adequately treated brain metastases were allowed. No imbalances were reported in either arms regarding brain metastases (~12%), histology (~24% of squamous), ECOG performance status (~30% PS0), or current smokers (~20% in both arms). A higher proportion of females was included in the chemotherapy arm (45.2% versus 32.1%). Maintenance treatment was prescribed in 38% of patients [31]. No benefit was seen with nivolumab compared to chemotherapy in terms of the primary endpoints PFS (4.2 versus 5.9 months, HR 1.15, 95% CI 0.91–1.45, *p* = 0.251), OS (14.4 versus 13.2 months, HR 1.02, 95% CI 0.80–1.30), or RR (26.1% versus 33.5%). However, the toxicity profile favored nivolumab, with 17.6% of patients having grade 3–4 AEs compared with 50.6% in the chemotherapy arm (Table 1). Of note, patients with NSCLC with strong PD-L1 expression (TPS ≥ 50%) did not derive a greater benefit from nivolumab than those with weaker expression. Nivolumab was the post-discontinuation treatment in 60% of the patients in the chemotherapy arm. The lack of survival benefit could be related to various hypothetical factors. First, there was a higher proportion of tumors with strong PD-L1 expression (TPS ≥ 50%) in the control arm compared to the nivolumab arm (74.1% versus 53.2%). Second, only 44% of patients in the nivolumab arm received second-line treatment, mostly platinum-based chemotherapy, suggesting that a certain subgroup of patients was untreated [31]. This could be a consequence of hyper-progressive diseases on immunotherapy, as recently reported by Champiat et al. [32]. Overall, results from Check-Mate 26 in the whole population and for those tumors with strongly positive PD-L1 expression are inconsistent with first-line nivolumab performance in phase I/II trials [33].

Although the reason for these contrasting results between KEYNOTE 024 [15] and Checkmate 026 [31] trial remains unclear, we should considered the nivolumab trial as negative, and we believe that differences in patient selection are the primary causes of this discrepancy. Differences in biomarker tests and in PD-L1 expression cut-off point (22C3 and 50% with pembrolizumab versus 28–8 clone and 5% with nivolumab) could have contributed to the discordant results between the trials. This means that patients with strong PD-L1 positivity in the KEYNOTE trial may not be similar to patients with strong PD-L1 positivity in the Checkmate trial, because the sensitivity of the relevant clones used to define PD-L1 status is potentially different. Additionally, PD-L1 testing was performed after metastatic diagnosis in the pembrolizumab trial, whereas in the nivolumab trial it was performed in archival tissue biopsy specimens taken within 6 months prior to randomization. However, in the KEYNOTE 010 trial, survival benefit with pembrolizumab as a second-line treatment was independent of whether the PD-L1 test was performed in an archival or in a new tissue biopsy specimen [11]. The efficacy of immune checkpoint inhibitors is higher among smokers [16]. A higher percentage of never-smoker patients was included in the nivolumab trial than in the pembrolizumab trial (11% versus 3%); such patients have lower mutational loads that negatively correlate with the success of immune checkpoint-targeting therapies [34]. Another major difference between the trials was the percentage of patients who received prior radiotherapy and were enrolled in the trial. This percentage was abnormally high (37.6%) for patients enrolled in the CheckMate 026 trial [31]. In the KEYNOTE 024 [15] trial, prior radiation therapy of >30 Gy within 6 months of the first dose of trial treatment was an exclusion criterion. Therefore, sites that were involved in both trials may have operationally favored enrolment of all previously irradiated patients in to Checkmate026. It is clear that previous radiotherapy can have major consequences on the tumor microenvironment [35] and potentially lead to decreased activity of immune checkpoint inhibitors in previously irradiated areas. At this stage it is unknown which areas (mediastinum, others) were previously irradiated in patients enrolled in the Checkmate 026 trial [31].

Other first-line randomized phase III clinical trials are testing anti-PD-1 monotherapy, such as nivolumab in CheckMate 227 (NCT02477826), or anti-PD-L1 monotherapies, such as atezolizumab in IMpower 110 (NCT02409342) and avelumab in the ongoing JAVELIN Lung 100 trial (NCT02576574). These trials may validate immune checkpoint inhibitors as a first-line treatment in patients with PD-L1-positive NSCLC.

## Combination of anti-PD-1 antibodies with chemotherapy

A large body of preclinical data has shown that chemotherapy and radiation modulate the immune response against tumors [36], and that chemotherapy can induce PD-L1 expression in tumor cells [37, 38],. This has led to clinical investigation of combinations of immune checkpoint inhibitors and chemotherapy.

The phase II KEYNOTE-021 trial (*n* = 123) compared pembrolizumab 200 mg for 2 years concomitant with four cycles of carboplatin-pemetrexed chemotherapy followed by pemetrexed as maintenance therapy with chemotherapy alone [39]. A higher proportion of never-smoker patients (25% versus 14%) and patients with adenocarcinoma histology (97% versus 87%) were included in the pembrolizumab arm.

A total of 32% of patients in the chemotherapy group crossed over to receive pembrolizumab monotherapy as allowed by the study protocol. The combination arm had improved RR (55% versus 29%, *p* = 0.0016, with 80% RR among strongly PD-L1-positive tumors) and PFS (13.0 months versus 8.9 months, HR 0.53, 95% CI 0.31–0.91, *p* = 0.010) compared to chemotherapy alone; however, the frequency of grade 3–4 treatment-related AEs was higher in the concomitant arm (39% versus 26%; Table 1). Of note, the chemotherapy arm also demonstrated impressive PFS, suggesting a high level of patient selection. Indeed, median PFS was much longer than in similar populations included in other trials with the same chemotherapy schedule, such as the POINTBREAK trial (5.6 months) [40]. The KEYNOTE 021 study [39] reported a shorter time to response in the combination arm compared to the chemotherapy alone arm (1.5 months versus 2.7 months), suggesting this approach could be a good strategy for symptomatic patients [39]. These results are similar to those previously reported in phase I trials, suggesting that combination treatment could be an optimal strategy.

Overall, while very promising, these results need to be validated in a phase III trial. The preliminary RR of 80% in patients with tumors harboring PD-L1 expression ≥50% treated with the combination therapy appears intriguing, but numbers are too small to draw any definitive conclusions. The ongoing phase III trials KEYNOTE-189 (NCT02578680) and KEYNOTE-407 (NCT02775435) with pembrolizumab; and the IMpower 132 (NCT02657434), IMpower 130 (NCT02367781), IMpower 131 (NCT02367794), and IMpower 150 (NCT02366143) trials with atezolizumab are testing combination treatment versus standard of care and could help clarify the best treatment strategy for this population.

## Other open questions in the in first-line setting

**Fig. 1:**
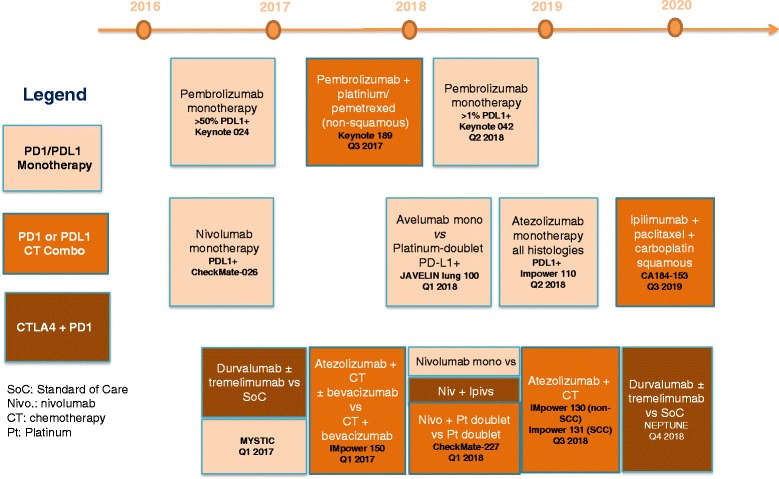
Upcoming randomized immunotherapy trials in first-line NSCLC and projected read-out timelines

The third approach to position immunotherapy in the first-line setting is the combination of PD-1/PD-L1 blockade with anti-cytotoxic T-lymphocyte-associated protein 4 (CTLA-4) compounds (Fig. 1).

Early preclinical studies have suggested that combined CTLA-4 and PD-1 pathway blockade produces synergistic anti-tumor activity [5, 41], providing the rationale for clinical studies. The high efficacy of nivolumab plus ipilimumab has been recently reported in PD-L1-positive tumors [42] (Table 1). The phase III CheckMate 227 (NCT02477826) trial with nivolumab plus ipilimumab, and the MYSTIC (NCT02453282) and NEPTUNE (NCT02542293) trials with durvalumab plus tremelimumab are comparing this strategy to anti-PD-1/PD-L1 monotherapy or chemotherapy. The toxicity profiles of these combinations might, however, limit their applicability.

The optimal strategy for NSCLC patients with tumor PD-L1 expression <50% has to be better defined: potential candidate therapies include concomitant treatments with chemotherapy, a combination of immune checkpoint inhibitors, or sequential strategies. This issue is important because of the limited standard second-line options currently available [43, 44] in cases in which immune checkpoint inhibitors are prescribed as the first-line treatment.

The treatment duration with immune checkpoint inhibitors is an important issue as well as economic costs. Therefore, detailed health economic analysis are needed to avoid inequities to access for these treatments [45]. New tools should be applied, such as the ESMO Magnitude of Clinical Benefit Scale (ESMO-MCBS), which uses a rational, structured, and consistent approach to derive a relative ranking of the magnitude of clinically meaningful benefit that can be expected from new anti-cancer therapies [46, 47].

## Conclusions

Immune checkpoint inhibitors are the standard of care for second-line treatment in advanced NSCLC, and pembrolizumab should be considered a standard first-line treatment in NSCLC patients with a good PS whose tumors have PD-L1 expression ≥50%. PD-L1 status determined by immunohistochemistry should be considered a reflex biomarker, along with *EGFR* mutation and *ALK* translocation, for guiding treatment of front-line patients with advanced NSCLC.

Discrepancies in patient selection (notably, previous radiotherapy) and PD-L1 testing methods could explain the negative results achieved with nivolumab in the first-line setting. Better outcomes were observed with chemotherapy combined with pembrolizumab compared to chemotherapy alone in a small-randomized phase II trial. Whether this strategy is better than immunotherapy alone or the combination of different checkpoint inhibitors remains unknown, because no phase III trial data is yet available.
