# Erratum to: Successes and failures: what did we learn from recent first-line treatment immunotherapy trials in non-small cell lung cancer?

**DOI:** 10.1186/s12916-017-0857-x

**Published:** 2017-04-20

**Authors:** Jordi Remon, Benjamin Besse, Jean-Charles Soria

**Affiliations:** 1 Cancer Medicine Department, Gustave Roussy, Villejuif, France; 2 Medical Oncology Department, Hospital Vall d’Hebron, Barcelona, Spain; 3 University Paris-Sud, Orsay, France

## Erratum

After publication of the original article [1], it came to the publisher’s attention that the text contained several minor typos. Upon investigation, it transpired that there had been technical issues affecting the copyediting process before the article entered the production stage, resulting in the presence of minor errors, without influence on content, in the published version.

The original article has now been updated in order to rectify these errors, comprising of textual edits that do not affect the scientific content of the paper.
